# The impact of design elements on undergraduate nursing students’ educational outcomes in simulation education: protocol for a systematic review

**DOI:** 10.1186/s13643-022-01926-3

**Published:** 2022-03-23

**Authors:** Matthew Jackson, Lauren McTier, Laura A. Brooks, Rochelle Wynne

**Affiliations:** 1 School of Nursing & Midwifery, Deakin University, 1 Gheringhap Street, Geelong, VIC Australia; 2 Centre for Quality & Patient Safety Research, Deakin University, 1 Gheringhap Street, Geelong, VIC Australia; 3 Institute for Health Transformation, Deakin University, 1 Gheringhap Street, Geelong, VIC Australia; 4 Western Sydney Nursing & Midwifery Research Centre, Blacktown Clinical & Research School, Western Sydney University & Western Sydney Local Health District, Blacktown Hospital, Marcel Crescent, Blacktown, NSW Australia; 5 School of Nursing & Midwifery, Western Sydney University, Locked Bag, Penrith, NSW 1797 Australia

**Keywords:** Nursing, Simulation, Undergraduate, Student, Systematic review, Protocol

## Abstract

**Background:**

Although simulation-based education (SBE) has become increasingly popular as a mode of teaching in undergraduate nursing courses, its effect on associated student learning outcomes remains ambiguous. Educational outcomes are influenced by SBE quality that is governed by technology, training, resources and SBE design elements. This paper reports the protocol for a systematic review to identify, appraise and synthesise the best available evidence regarding the impact of SBE on undergraduate nurses’ learning outcomes.

**Methods:**

Databases to be searched from 1 January 1990 include the Cumulative Index to Nursing and Allied Health Literature (CINAHL), the Medical Literature Analysis and Retrieval System Online (MEDLINE), American Psychological Association (APA) PsycInfo and the Education Resources Information Centre (ERIC) via the EBSCO host platform. The Excerpta Medica database (EMBASE) will be searched via the OVID platform. We will review the reference lists of relevant articles for additional citations. A combination of search terms including ‘nursing students’, ‘simulation training, ‘patient simulation’ and ‘immersive simulation’ with common Boolean operators will be used. Specific search terms will be combined with either MeSH or Emtree terms and appropriate permutations for each database. Search findings will be imported into the reference management software (Endnote© Version.X9) then uploaded into Covidence where two reviewers will independently screen the titles, abstracts and retrieved full text. A third reviewer will be available to resolve conflicts and moderate consensus discussions. Quantitative primary research studies evaluating the effect of SBE on undergraduate nursing students’ educational outcomes will be included. The Mixed Methods Appraisal Tool (MMAT) will be used for the quality assessment of the core criteria, in addition to the Cochrane RoB 2 and ROBINS-I to assess the risk of bias for randomised and non-randomised studies, respectively. Primary outcomes are any measure of knowledge, skills or attitude.

**Discussion:**

SBE has been widely adopted by healthcare disciplines in tertiary teaching settings. This systematic review will reveal (i) the effect of SBE on learning outcomes, (ii) SBE element variability and (iii) interplay between SBE elements and learning outcome. Findings will specify SBE design elements to inform the design and implementation of future strategies for simulation-based undergraduate nursing education.

**Systematic review registration:**

PROSPERO CRD42021244530

**Supplementary Information:**

The online version contains supplementary material available at 10.1186/s13643-022-01926-3.

## Background

Simulation-based education (SBE) is a popular mode of teaching in undergraduate nursing education [1]. This pedagogical approach provides students with an opportunity to practise non-technical skills (e.g. communication, teamwork, problem solving) and clinical skills (e.g. venepuncture), in a controlled environment that poses no risk of harm to patients and allows the possibility of direct feedback and guidance from teaching staff [2]. Educational outcomes are influenced by SBE quality that is governed by technology, training, resources and SBE design elements [3]. As SBE has evolved, its design elements have been adjusted to overcome learning barriers such as excessive student anxiety [4]. SBE has also been tailored to suit a variety of settings, populations, time frames and session frequency [5].

SBE designs include tag team simulation, simulated virtual and augmented environments and game-based themes within the traditional simulation, to accommodate ever-expanding university enrolments whilst maintaining student experience and satisfaction [6]. In the context of evolving SBE, the level of evidence for SBE effectiveness in nursing education is diverse, and educational outcomes remain ambiguous [7]. SBE can refer to a low-fidelity task trainer that takes 5 min to complete a skill whilst working one-on-one with a facilitator, or a complex high-fidelity immersive scenario involving a group of students performing complex tasks over a prolonged period of time [8]. The pedagogy of SBE generally fails to acknowledge the complex variability in design elements [9] that has implications for learning outcomes. As such, there is a need to evaluate the effect of SBE on learning and to describe SBE design elements to determine whether specific elements have a superior effect on learning outcomes. The aim of this systematic review is to identify, appraise and synthesise the best available evidence regarding the effectiveness of SBE and the impact of design elements on undergraduate nurses’ learning outcomes. To address this aim, there are three review objectives:

(i) To determine the effect of SBE on learning outcomes
(ii) To describe the variability in SBE elements
(iii) To explore the interplay between SBE elements and learning outcomes

## Methods

### Design

This systematic review protocol was developed in accordance with the Preferred Reporting Items for Systematic Review and Meta-Analysis Protocols (PRISMA-P) [10] statement (Additional file 1) and the accompanying elaboration and explanation guide [11]. The planned systematic review will be reported in accordance with the PRISMA statement [12] and registered on the International Prospective Register of Systematic Reviews (PROSPERO) (CRD42021244530).

### Eligibility criteria

Study selection will be guided by the eligibility criteria and the Population, Intervention, Comparison, Outcome (PICO) pneumonic [13].

### Types of studies

Primary quantitative research studies published in English from 1 January 1990 will be considered. There has been a sustained increase in the volume of research focused on SBE since the 1990s related to the affordability of high-fidelity simulators [5]. Relevant quantitative designs including randomised controlled trials and quasi-experimental studies with a control group for comparison will be included.

### Population

The population of interest is undergraduate nursing students, aged 18 years or over, engaged in SBE in an academic setting, such as a university.

### Intervention

SBE is the intervention of interest. In general terms, SBE is a teaching technique used to enhance a learning setting to appear like the real world. In healthcare, there are a variety of design elements that can impact the effectiveness of SBE that in undergraduate nursing education settings is usually conducted in a physical environment with face-to-face teaching. Elements include devices such as manikins or task trainers that are used to allow students to interact in a manner that represents real clinical practice.

Fidelity refers to the realism of the simulation environment [14], and low, medium or high-fidelity environments are elements of simulation that can influence learning outcomes. Simulated patients, including trained actors, and/or integrated simulators that incorporate technology influence fidelity and as such the realism of the interaction between the learner and the manikin. In addition, adjustment of the manikin’s physiological data based on learner decision-making or conversing with the learner through built-in speakers to simulate a patient conversation [15] is a common element. Task trainers are another element of simulation designed to mimic a part of the patient’s body such as a manikin arm for intravenous insertion or something as simple as an orange, to practise injections.

### Comparator

Conventional education modalities such as didactic lectures, passive (one-way) classroom teaching or small group seminars will provide a comparative control cohort.

### Outcome

The primary outcome will be the SBE effect. This effect will be measured by assessing at least one measure of knowledge, skills or attitude as an endpoint. For the purposes of this review, knowledge is defined as learnt information (e.g. theoretical knowledge relating to the intended learning outcome of the simulation activity) acquired within the simulation activity. The measurement of knowledge will be determined by an academic outcome assessment. Skills are defined as the ability to develop psychomotor function aligned with the successful performance of a particular procedure (e.g. change a wound dressing), and attitude is defined as how worthwhile the learner believes the activity is in relation to their learning [16].

### Exclusion criteria

Given the primary aim of the review is to assess the effect of SBE, which necessitates the need for a comparator group, case-control, cohort, cross-sectional and single-group observational studies will be excluded. Literature, narrative, integrative mixed methods and systematic reviews, observational cohort studies, abstracts, letters, commentary, editorials, opinion pieces and grey literature will be excluded. SBE can use modified realities such as augmented reality and virtual reality. These emerging digital modalities have a limited body of evidence and are a developing area of practice [17] so beyond the scope of this review. The use of pre-simulation interventions such as online learning modules or smart device technologies may influence the relationship between SBE and traditional learning so will be excluded. Similarly, studies that combine low-fidelity SBE elements with traditional education approaches and compare these to medium- or high-fidelity SBE will be excluded. Postgraduate nursing students, midwifery students or students receiving SBE in a clinical setting (e.g. a hospital) are excluded. Manuscripts published in languages other than English will be excluded. This is an unfunded study, so translation costs are beyond the investigator’s capacity.

### Information sources and search strategy

Databases to be searched from 1 January 1990 include the Cumulative Index to Nursing and Allied Health Literature (CINAHL), the Medical Literature Analysis and Retrieval System Online (MEDLINE), American Psychological Association (APA) PsycInfo and the Education Resources Information Centre (ERIC) via the EBSCO host platform. The Excerpta Medica database (EMBASE) will be searched via the OVID platform. The MEDLINE search strategy is included in Table 1.

**Table 1:** EBSCO MEDLINE search strategy

	MeSH headings and search terms
1	MH “students, nursing”
2	MH “Education, Nursing, baccalaureate”
3	Undergrad*
4	College*
5	Student*
6	University*
7	1 OR 2 OR 3 OR 4 OR 5 OR 6
8	MH “Simulation training+”
9	Simulation N3 training
10	Simulation N3 education
11	Simulation N3 patient
12	8 OR 9 OR 10 OR 11
13	Nurs*
14	7 AND 12 AND 13

*The symbol is used to explode key search terms in MEDLINE as explained in the methods.

Primary quantitative studies included in systematic reviews captured by the search strategy and studies identified through secondary searching relevant study reference lists will also be eligible for inclusion. Specific search terms will be developed in MEDLINE with assistance from a senior librarian experienced in the conduct of systematic reviews, using text words and subject headings. Primary search terms include ‘nursing students’, ‘simulation training, ‘patient simulation’ and ‘immersive simulation’ with common Boolean operators and symbols for exploding terms (*, +) as illustrated in Table 2. Each database will be searched using these broad terms with either MeSH or Emtree terms with appropriate permutations.

**Table 2:** Search concepts

Concept 1	Concept 2	Concept 3
MH “students, nursing”	MH “Simulation training+”	Nurs*
OR	OR	
MH “Education, Nursing, baccalaureate”	Simulation N3 training	
OR	OR	
Undergrad*	Simulation N3 education	
OR	OR	
College*	Simulation N3 patient	
OR		
Student*		
OR		
University*		

Each concept will be combined with “AND”

*The symbol is used to explode key search terms in MEDLINE as explained in the methods

### Data management, selection and screening

Database search results will be imported into the reference management software Endnote© VersionX9 and uploaded into Covidence. Covidence is a tool for effective collaborative title and abstract screening, full-text screening, data abstraction and quality assessment [18]. Covidence automatically identifies, sorts and removes duplicate studies. Two reviewers (MJ and RW) will independently screen the title and abstract of citations with a third reviewer (LM) available to moderate disagreements with a view to reaching consensus. Articles that meet the eligibility criteria will be sourced for full-text review. Two reviewers (MJ and RW) will complete the full-text screening with a third reviewer (LM) available to moderate disagreements to achieve consensus. Screening outcomes will be documented and reported in a PRISMA flow diagram [12].

### Data extraction

Full-text data extraction will be undertaken in duplicate by two reviewers (MJ and LB) and conflicts resolved with arbitrary discussion. Data extraction will take place using a modified version of the existing Covidence data extraction template. Study characteristics to be recorded include publication details (authors, publication year), demographic characteristics, methodology, intervention and comparator group details and reported outcomes. To ensure consistency between reviewers, periodic meetings will be concurrently held during the screening process to resolve disagreements by discussion. A third reviewer (LM) will act as an adjudicator in the event of an unresolved agreement.

### Data items

The following data items will be extracted from the selected studies: study setting, sample, inclusion and exclusion criteria, aim, design element/s employed, unit of allocation, start and end dates of the study, duration of participation, baseline group differences, frequency and duration of the intervention, outcome measured (e.g. knowledge acquisition; skill improvement, attitude and satisfaction), tool used to measure outcome, validity of the tool/s, comparator group education method and sustainability of outcome/s.

### Outcomes and prioritisation

The primary outcome is the SBE effect measured by assessing at least one measure of knowledge, skills or attitude as an endpoint. Outcomes will be compared between the groups who do and do not participate in a SBE intervention. Secondary outcomes include describing variability in SBE design elements and sub-group analyses to explore the interplay between SBE elements and learning outcomes. In the case of discrepancies in the outcomes reported, contact with corresponding authors will be attempted by email to obtain relevant data.

### Risk of bias in individual studies

Each randomised trial will be assessed for possible risk of bias using the revised Cochrane Collaboration tool for assessing the risk of bias (RoB 2). This tool focuses on trial design, conduct and reporting to obtain the information relevant to the risk of bias [19]. Based on the answers provided within the tool, trials will be categorised as ‘low’ or ‘high’ risk of bias. Where a lack of detail impacts the capacity to assess the risk bias, this will be noted as ‘unclear’ and authors contacted for more information. If there is disagreement, a third author will be consulted to act as an arbitrator. The Risk Of Bias In Non-Randomized Studies – of Interventions (ROBINS-I) tool will be used for bias assessment in studies with this design. The ROBINS-I tool shares many features with the RoB 2 tool as it focuses on a specific result, is structured into a fixed set of domains of bias, includes signalling questions that inform risk of bias judgements and leads to an overall risk-of-bias judgement [20].

### Quality appraisal

Two authors (MJ and LB) will independently assess each article for quality using the Mixed Methods Appraisal Tool (MMAT) [21]. This critical appraisal tool allows the appraisal of five categories of studies including qualitative research, randomised controlled trials, non-randomised studies, quantitative descriptive studies and mixed methods studies. As recommended, each study will be reviewed by two authors by completing the appropriate study categories identified within the MMAT. An overall score will not be used to rate the quality of study, but instead, sensitivity analyses will be completed to determine the impact of study quality on outcomes by contrasting components as recommended [11].

### Data synthesis

The primary unit of analysis will reflect each endpoint measure; knowledge acquisition, skill improvement or attitude. Dichotomous outcomes will be extracted and analysed using odds ratio (OR) with 95% confidence interval (CI), and continuous outcomes will be represented using the mean difference (MD) or the standardised mean difference (SMD) when outcomes are measured using different scales, with 95% CI. In the event of missing data, we will attempt to contact the primary authors to obtain relevant information. Meta-analysis will be undertaken if two or more studies with comparable design and outcome measures meet the eligibility criteria. Pooled data will be analysed using the DerSimonian and Laird method for random-effects models in RevMan [22]. This model is used when reported outcome effects differ amongst studies but follow some similar distribution [23]. Findings from meta-analysis will be illustrated using forest plots.

The *I*^2^ test of heterogeneity will be used to determine the level of variation related to diversity rather than chance. Rather than simply stating whether heterogeneity is present or not, it will be measured using the *I*^2^ test [24]. Low heterogeneity will be reflected as an *I*^2^ result between 0 and 40%, moderate heterogeneity between 30 and 60%, substantial heterogeneity between 50 and 90% and considerable heterogeneity between 75 and 100% [25]. If a high heterogeneity level (*I*^2^ > 50%) amongst trials exists, study design and characteristics will be reported and sensitivity analyses conducted to reduce variability with a view to being able to undertake meta-analysis. It is assumed that specific design elements will underpin the need for subgroup analyses. If data are not suitable for meta-analysis, findings will be presented descriptively in the form of a narrative synthesis according to categories outlined in the SWiM guideline [26]. Due to the potential of a large amount of data that could be conveyed textually, the content will be sequenced to follow the same structure to ensure consistency across results [27].

### Meta-bias

Study selection for this review will be guided by the PICO pneumonic and the framework outlined in this protocol to reduce the risk of bias. Hand searching relevant systematic reviews will contribute to a reduction in publication bias [28]. To reduce bias associated with selecting studies, two independent reviewers will be used throughout the screening and data collection process [29]. To address bias when synthesising studies, this protocol has been registered prospectively to promote transparency and replicability [10]. Two reviewers will appraise the quality of studies, and low-quality studies will be excluded to avoid inappropriate influence on results [10].

### Confidence in cumulative evidence

The overall effectiveness of simulation and the impact of design elements on undergraduate nurses will be assessed with the Grades of Recommendation, Assessment, Development and Evaluation Working Group (GRADE) approach. The GRADE approach allows an assessment of the certainty of the body of evidence for each individual outcome [30]. Quality of evidence is dependent on the within-study risk of bias, directness of evidence, heterogeneity, precision of effect estimates and risk of publication bias [23]. Two authors will independently assess the quality of evidence regarding the effectiveness of simulation on each outcome. All disagreements will be resolved through discussion and consensus. The certainty of evidence from pooled analyses will be categorised as high, moderate, low or very low [30]. If data are not suitable for pooled meta-analysis and studies are grouped for synthesis, the SWiM guideline [26] will provide a framework for narrative analysis and GRADE criteria will be used to examine the certainty of the evidence for each analytical grouping.

## Discussion

SBE is seen as a panacea to counteract the potential for inadequate exposure to real-life clinical scenarios. It has become an increasingly popular mode of teaching yet reports of its effectiveness remain diverse. There is a variety of simulation designs, and embedded elements have implications for learning outcomes that are not well described or scrutinised in the literature. This systematic review will provide a detailed summary of evidence for SBE and SBE design elements in the context of undergraduate nursing education. Findings will contribute to the body of literature examining specific design elements by establishing the effect of SBE on learning outcomes and demonstrating the degree of variability in these elements.

## Supplementary Information

**Additional file 1.** PRISMA-P 2015 Checklist.

## Data Availability

Datasets generated by searches will be made available upon reasonable request.

## Abbreviations

SBE: Simulation-based education
CINAHL: Cumulative Index to Nursing and Allied Health Literature
MEDLINE: Medical Literature Analysis and Retrieval System Online
APA: American Psychological Association
ERIC: Education Resources Information Centre
EMBASE: Excerpta Medica database
MMAT: Mixed Methods Appraisal Tool
PROSPERO: Prospective Register of Systematic Reviews
PICO: Population, Intervention, Comparison, Outcome
